# Prevalence and Significance of AGR2 Expression in Human Cancer

**DOI:** 10.1002/cam4.70407

**Published:** 2024-11-12

**Authors:** Nina Schraps, Jacob Constantin Port, Anne Menz, Florian Viehweger, Seyma Büyücek, David Dum, Ria Schlichter, Andrea Hinsch, Christoph Fraune, Christian Bernreuther, Martina Kluth, Claudia Hube‐Magg, Katharina Möller, Viktor Reiswich, Andreas M. Luebke, Patrick Lebok, Sören Weidemann, Guido Sauter, Maximilian Lennartz, Frank Jacobsen, Till S. Clauditz, Andreas H. Marx, Ronald Simon, Stefan Steurer, Baris Mercanoglu, Nathaniel Melling, Thilo Hackert, Eike Burandt, Natalia Gorbokon, Sarah Minner, Till Krech, Florian Lutz

**Affiliations:** ^1^ General, Visceral and Thoracic Surgery Department and Clinic University Medical Center Hamburg‐Eppendorf Hamburg Germany; ^2^ Institute of Pathology, University Medical Center Hamburg‐Eppendorf Hamburg Germany; ^3^ Institute of Pathology, Clinical Center Osnabrueck Osnabrueck Germany; ^4^ Department of Pathology Academic Hospital Fuerth Fuerth Germany

## Abstract

**Backround:**

Anterior gradient 2 (AGR2) is a resident endoplasmic reticulum (ER) protein with a vital role in embryonal development, mucus maturation, tissue regeneration, and wound healing.

**Methods:**

To determine the prevalence and clinical significance of AGR2 expression in cancer, a tissue microarray containing 14,966 tumors from 134 different tumor types and subtypes as well as 608 samples of 76 different normal tissue types was analyzed by immunohistochemistry (IHC).

**Results:**

AGR2 positivity was found in 103 of 134 tumor categories, and 83 tumor categories contained at least one strongly positive case. AGR2 expression was most frequently seen in tumors of the female genital tract, particularly adenocarcinomas (up to 100%), various breast cancer subtypes (57.1%–100%), urothelial carcinoma (74.6%–100%), adenocarcinomas of the upper and lower gastrointestinal tract (93.6%–99.6%), and pancreaticobiliary cancers (65.2%–98.2%). AGR2 positivity was slightly less common in squamous cell carcinomas (46.4%–77.3%) and mainly absent in mesenchymal and lymphoid tumors. While AGR2 expression was only weak or absent in the normal thyroid, it was moderate to strong in 46.0% of adenomas, 52.8% of follicular carcinomas, and 81.8% of papillary carcinomas of the thyroid. High AGR2 expression was strongly linked to poor ISUP (*p* < 0.0001), Fuhrman (*p* < 0.0001), and Thoenes (*p* < 0.0001) grades as well as advanced pT stage (*p* = 0.0035) in clear cell renal cell carcinoma (ccRCC). Low AGR2 expression was associated with high BRE grade in breast cancer (*p* = 0.0049), nodal metastasis (*p* = 0.0275) and RAS mutation (*p* = 0.0136) in colorectal cancer, nodal metastasis (*p* = 0.0482) in endometrioid endometrial carcinoma, high grade in noninvasive urothelial carcinoma (*p* = 0.0003), and invasive tumor growth in urothelial carcinoma (*p* < 0.0001).

**Conclusions:**

It is concluded that AGR2 expression occurs in a broad range of different tumor entities and that AGR2 assessment may serve as a diagnostic aid for the distinction of thyroidal neoplasms and as a prognostic marker in various cancer types.

## 1 Introduction

Anterior gradient 2, AGR2, is a resident endoplasmic reticulum (ER) protein with a role in oxidative protein folding in the endoplasmic reticulum [1]. The protein is also termed Xenopus Anterior gradient 2 (XAG2) or secreted cement gland protein XAG‐2 homolog because it was initially discovered in the African frog *Xenopus laevis*, where it plays a role in embryonic development and cement gland differentiation [1, 2]. AGR2 is not restricted to the ER, has protein disulfide isomerase (PDI) activity, and exerts physiological roles in embryonal development, mucus maturation, tissue regeneration, and wound healing [1, 3, 4, 5, 6]. Its mucus stabilizing role is critical for maintaining epithelial barrier function in the intestine, where AGR2 forms a heterodisulfide bond with cysteine residues of MUC2 [4, 7]. Disturbed AGR2 function causes intestinal pathology due to a disrupted mucin maturation [4, 7, 8]. *AGR*2 knockout mice show a loss of intestinal mucus and develop ileitis and colitis [8]. A critical role of AGR2 in gastrointestinal health is also supported by the observation of a human enteropathy caused by a loss‐of‐function variant of AGR2 which is associated with a disturbed processing of mucins, increased ER stress, and goblet cell loss [7].

Altered expression of AGR2 has been implicated with several signal transduction pathways and cell functions related to cancer initiation, progression, and metastasis [9, 10, 11, 12, 13]. For example, high AGR2 levels have been linked to downregulation of the p53 response [14], increased cell migration [13], and cell transformation in cancer cell lines [15] although others have observed repression of cell growth and proliferation in case of high AGR2 levels [16, 17]. In breast and prostate cancer, AGR2 expression has been linked to estrogen receptor [17, 18, 19] and androgen receptor regulation [20, 21]. Studies evaluating larger cohorts of cancers by immunohistochemistry (IHC) have identified associations between high AGR2 expression and unfavorable tumor phenotype or poor prognosis in adenocarcinoma of the lung [10], gastric adenocarcinoma [22], and adenocarcinoma of the prostate [23]. However, the number of studies analyzing AGR2 in cancer is still limited. In 34 studies using IHC, only 26 different tumor entities have so far been evaluated (Medline, May 2024). Moreover, in 12 cancer entities where multiple studies have been executed, the obtained results were sometimes considerably discrepant. For example, the range of reported AGR2‐positive cases ranged from 56.8% to 100% in adenocarcinoma of the lung [10, 24], from 57.0% to 95.4% in prostate cancer [21, 23], and from 19.0% to 46.1% in serous carcinoma of the ovary [13, 25]. Such conflicting results could be caused by the use of different antibodies, immunostaining protocols, and criteria to define positivity.

To better understand the prevalence and potential clinical significance of AGR2 expression in cancer, a comprehensive study analyzing a large number of neoplastic and nonneoplastic tissues under highly standardized conditions is needed. Therefore, AGR2 expression was analyzed in more than 14,000 tumor tissue samples from 134 different tumor types and subtypes as well as 76 nonneoplastic tissue categories by immunohistochemistry (IHC) in a tissue microarray (TMA) format in this study.

## 2 Materials and Methods

### 2.1 Tissue Microarrays (TMAs)

The normal tissue TMA was composed of eight samples from eight different donors for each of 76 different normal tissue types (608 samples on one slide). The cancer TMAs contained a total of 14,966 primary tumors from 134 tumor types and subtypes. Detailed histopathological data on grade, pT, or pN were available from subsets of colorectal cancer (*n* = 2351), endometrioid endometrial carcinoma (*n* = 182), clear cell (*n* = 1224) and papillary renal cell carcinoma (*n* = 310), serous (*n* = 369) and endometrioid carcinoma of the ovary (*n* = 40), thyroid carcinoma (*n* = 518), urothelial carcinoma (*n* = 829), as well as pancreatic carcinomas (*n* = 598). Clinical follow‐up data were available from 789 patients with ccRCC and from 177 patients with pRCC with a median follow‐up time of 48.0 and 50.5 months (range 1–250 and 1–247) and 254 patients with urothelial carcinoma with a median follow‐up time of 14.0 months (range 1–77). The composition of both normal and cancer TMAs is described in detail in the results section. All samples were from the archives of the Institutes of Pathology, University Hospital of Hamburg, Germany, the Institute of Pathology, Clinical Center Osnabrueck, Germany, and Department of Pathology, Academic Hospital Fuerth, Germany. Tissues were fixed in 4% buffered formalin and then embedded in paraffin. The TMA manufacturing process was described earlier in detail [26, 27]. In brief, one tissue spot (diameter: 0.6 mm) per patient was used. The use of archived remnants of diagnostic tissues for manufacturing of TMAs and their analysis for research purposes as well as patient data analysis has been approved by local laws (HmbKHG, §12) and by the local ethics committee (Ethics commission Hamburg, WF‐049/09). All work has been carried out in compliance with the Helsinki Declaration.

### 2.2 Immunohistochemistry (IHC)

Freshly cut TMA sections were immunostained on 1 day and in one experiment. Slides were deparaffinized with xylol, rehydrated through a graded alcohol series and exposed to heat‐induced antigen retrieval for 5 min in an autoclave at 121°C in pH 7.8 Tris‐EDTA‐Citrat (TEC) puffer. Endogenous peroxidase activity was blocked with Dako REAL Peroxidase‐Blocking Solution (Agilent Technologies, Santa Clara, CA, USA; #S2023) for 10 min. Primary antibody‐specific against AGR2 protein (rabbit recombinant, HMV‐325, ardoci GmbH, Hamburg, Germany) was applied at 37°C for 60 min at a dilution of 1:150. For the purpose of antibody validation, the normal tissue TMA was also analyzed by the rabbit recombinant AGR2 antibody EPR3278 (ab76473, Abcam Limited, Cambridge CB2 0AX, GB) at a dilution of 1:1800 and an otherwise identical protocol. Bound antibody was then visualized using the Dako REAL EnVision Detection System Peroxidase/DAB+, Rabbit/Mouse kit (Agilent Technologies, Santa Clara, CA, USA; #K5007) according to the manufacturer's directions. The sections were counterstained with hemalaun. For tumor tissues, the percentage of AGR2‐positive tumor cells was estimated, and the staining intensity was semi‐quantitatively recorded (0, 1+, 2+, 3+). For statistical analyses, the staining results were categorized into four groups as follows: negative: no staining at all, weak staining: staining intensity of 1+ in ≤ 70% or staining intensity of 2+ in ≤ 30% of tumor cells, moderate staining: staining intensity of 1+ in > 70%, staining intensity of 2+ in > 30% but in ≤ 70% or staining intensity of 3+ in ≤ 30% of tumor cells, strong staining: staining intensity of 2+ in > 70% or staining intensity of 3+ in > 30% of tumor cells. The evaluation was performed by an experienced pathologist (SB).

### 2.3 Statistics

Statistical calculations were performed with JMP17 software (SAS, Cary, NC, USA). Contingency tables and the chi‐squared test were performed to search for associations between AGR2 staining and tumor phenotype. The log‐rank test was applied to detect significant differences between groups.

## 3 Results

### 3.1 Technical Issues

A total of 12,434 (83.1%) of 14,966 tumor samples were interpretable in our TMA analysis. Noninterpretable samples demonstrated a lack of unequivocal tumor cells or a lack of entire tissue spots. A sufficient number of samples (≥ 4) of each normal tissue type was evaluated.

### 3.2 AGR2 Immunostaining in Normal Tissues

**FIGURE 1:**
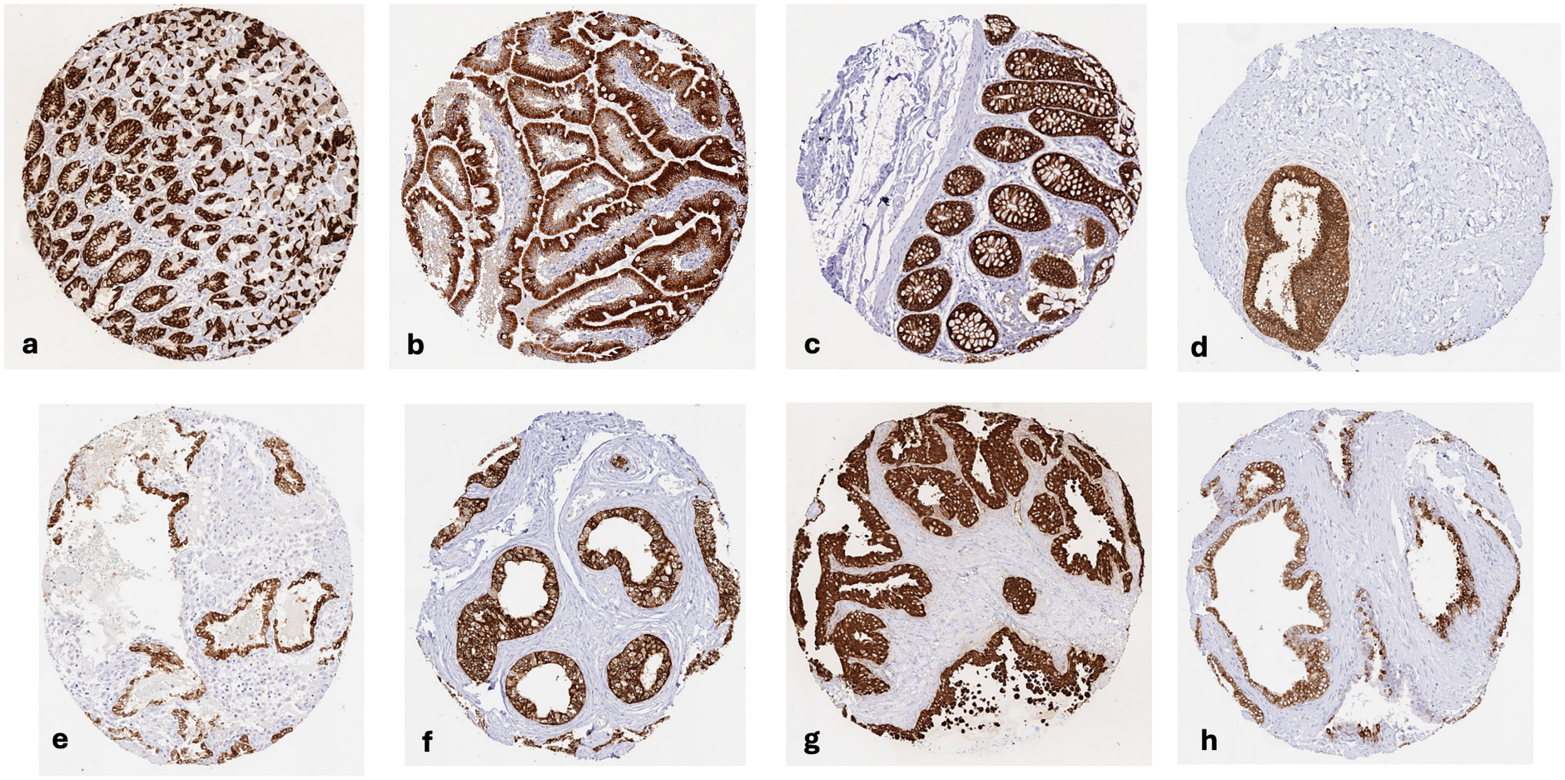
AGR2 immunostaining of normal tissues. The panels show a strong cytoplasmic AGR2 immunostaining of all epithelial cells of the stomach except parietal cells (a), as well as of all epithelial cells of the duodenal mucosa (b), the colorectal mucosa (c), the endocervical epithelium (d), the endometrium of the pregnant uterus (e), the cauda epididymis (f), and the seminal vesicle (g). Most epithelial cells of a prostate sample are also AGR2 positive (h).

A strong, predominantly cytoplasmic AGR2 staining occurred in various epithelial cell types such as from the colorectum, stomach (except parietal cells), gallbladder, pancreas (intercalated ducts and excretory ducts), seminal vesicle, urothelium, respiratory epithelium, lung (a large subset of pneumocytes), breast (a subset of luminal cells), endocervix, endometrium, and the fallopian tube. A somewhat less intense AGR2 staining also occurred in glandular (especially mucinous) and excretory duct cells of salivary glands, Brunner glands of the duodenum, a small subset of tubuli/collecting ducts of the kidney, a subset of epithelial cells in the epididymis, and a subset of epithelial cells of the adenohypophysis. Staining was variable ranging from absent to strong in pancreatic acinar cells as well as in acinar and basal cells of the prostate. AGR2 staining was mostly absent in squamous epithelial cells except in the tonsil, a fraction of cells of corpuscles of Hassall's, and the inner (Huxley) layers of hair follicles of the skin. In the thyroid, AGR2 staining was mostly absent although a weak to moderate staining occasionally was seen. Representative images are shown in Figure 1. All cell types found to be positive by HMV‐325 were also confirmed as AGR2 positive by EPR3278 (Figure S1). AGR2 staining was always absent in hepatocytes, testis, ovary, placenta, adrenal gland, parathyroid, brain, mesenchymal tissues, and in hematolymphatic cells.

### 3.3 AGR2 Immunostaining in Neoplastic Tissues

**FIGURE 2:**
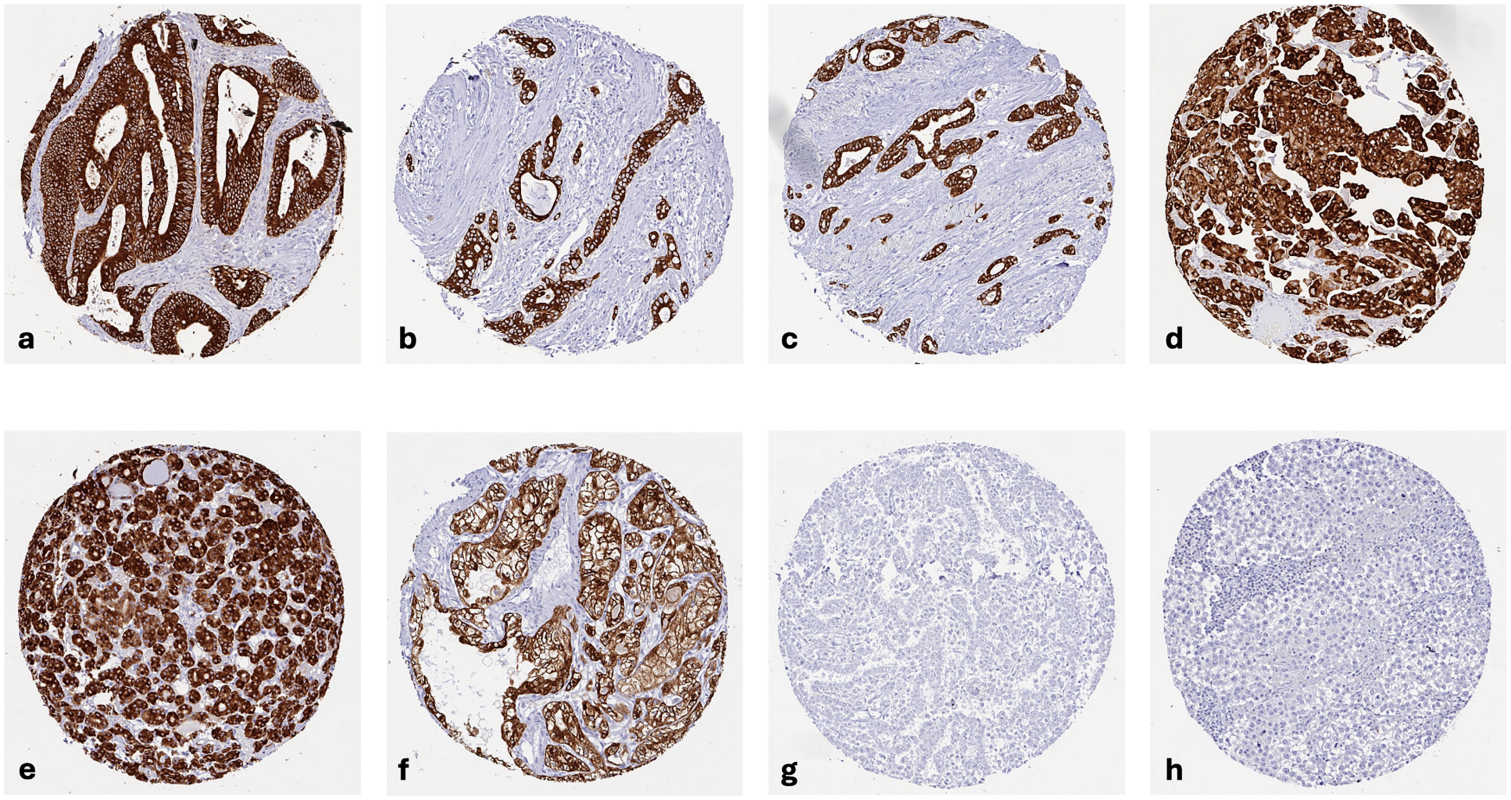
AGR2 immunostaining in cancer. AGR2 staining was cytoplasmic. The panels show a strong AGR2 positivity in cancer cells of adenocarcinoma of colon (a), gastric adenocarcinoma (b), adenocarcinoma of the pancreas (c), urothelial carcinoma (d), follicular thyroid carcinoma (e) and clear cell renal cell carcinoma (f), AGR2 immunostaining is absent in mesothelioma (g), and seminoma (h).

**FIGURE 3:**
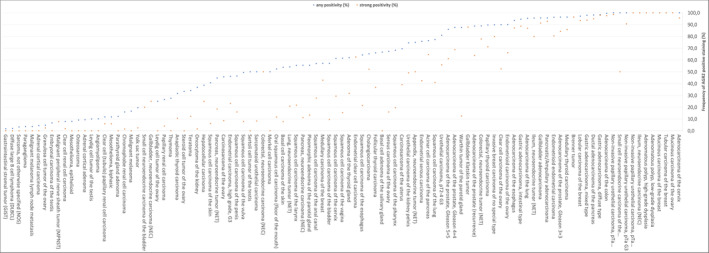
Ranking order of AGR2‐positive immunostaining in different human tumors. Orange dots show the percentage of strongly stained samples, whereas blue dots show the percentage of positive samples of any intensity.

A cytoplasmic AGR2 immunostaining was observed in 7514 (60.4%) of 12,434 analyzable tumors including 732 (5.9%) with weak, 874 (7.0%) with moderate, and 5908 (47.5%) with strong staining intensity. We did not see any membranous or extracellular staining of AGR2. Representative images are shown in Figure 2. At least an occasional weak AGR2 positivity was detected in 103 of 134 tumor types and tumor subtypes and 83 entities included at least one case with strong AGR2 positivity (Table 1). AGR2 expression was most frequently seen in tumors of the female genital tract, particularly adenocarcinoma of the cervix (100%) and mucinous carcinoma of the ovary (100%), various subtypes of breast cancer (57.1%–100%), urothelial carcinoma (74.6%–100%), adenocarcinoma of the upper and lower gastrointestinal tract (93.6%–99.6%), as well as in pancreatico‐biliary cancers (65.2%–98.2%). AGR2 positivity was slightly less common in neuroendocrine neoplasms (20.0%–100%) and in squamous cell carcinoma (46.4%–77.3%). AGR2 staining was only rarely seen in adrenocortical tumors (4.3%–9.3%) and in renal cell carcinoma (7.8%–26.5%), and mainly absent in melanoma, seminoma, embryonal carcinoma, adrenocortical neoplasms, tumors of the hematopoietic and lymphoid tissues, as well as mesenchymal tumors. A graphical representation of a ranking order of AGR2 positive and strongly positive cancers is given in Figure 3. The relationship between AGR2 expression and tumor phenotype in different cancer types is summarized in Table 2. High AGR2 expression was strongly linked to poor ISUP (*p* < 0.0001), Fuhrman (p < 0.0001), and Thoenes (p < 0.0001) grades as well as advanced pT stage (*p* = 0.0035) in clear cell renal cell carcinoma (ccRCC). AGR2 staining was associated with shortened recurrence‐free survival (*p* = 0.0275) in ccRCC. That AGR2 staining was unrelated to overall survival in ccRCC (*p* = 0.1826), may be due to the low number of positive cases (Figure S2). A reduced AGR2 expression was significantly associated with high BRE grade in breast cancer (*p* = 0.0049), nodal metastasis (p = 0.0275) and RAS mutation (*p* = 0.0136) in colorectal cancer, nodal metastasis (*p* = 0.0482) in endometrioid endometrial carcinoma, high grade in noninvasive urothelial carcinoma (*p* = 0.0003), and with invasive tumor growth in urothelial carcinoma (*p* < 0.0001). In urothelial carcinoma, AGR2 staining was unrelated to overall and recurrence‐free survival (*p* > 0.1). AGR2 immunostaining was unrelated to parameters of tumor aggressiveness in pancreatic, ovarian, and thyroidal cancer as well as papillary renal cell carcinoma although tumors with lower AGR2 expression often tended to exhibit more unfavorable tumor features.

**TABLE 1:** AGR2 immunostaining in human tumors.

	Tumor entity	On TMA (*n*)	AGR2 immunostaining				
			Analyzable (*n*)	Negative (%)	Weak (%)	Moderate (%)	Strong (%)
Tumors of the skin	Basal cell carcinoma of the skin	41	13	46.2	23.1	23.1	7.7
	Squamous cell carcinoma of the skin	95	81	59.3	23.5	11.1	6.2
	Malignant melanoma	19	18	83.3	16.7	0.0	0.0
	Malignant melanoma lymph node metastasis	86	83	96.4	2.4	1.2	0.0
	Merkel cell carcinoma	2	2	50.0	0.0	50.0	0.0
Tumors of the head and neck	Squamous cell carcinoma of the larynx	109	92	44.6	17.4	16.3	21.7
	Squamous cell carcinoma of the pharynx	60	41	31.7	26.8	22.0	19.5
	Oral squamous cell carcinoma (floor of the mouth)	130	103	47.6	30.1	10.7	11.7
	Pleomorphic adenoma of the parotid gland	50	45	44.4	15.6	26.7	13.3
	Warthin tumor of the parotid gland	49	48	12.5	33.3	41.7	12.5
	Basal cell adenoma of the salivary gland	15	15	33.3	26.7	40.0	0.0
Tumors of the lung, pleura, and thymus	Adenocarcinoma of the lung	196	130	4.6	3.1	5.4	86.9
	Squamous cell carcinoma of the lung	80	44	22.7	20.5	15.9	40.9
	Mesothelioma, epithelioid	40	25	92.0	0.0	8.0	0.0
	Mesothelioma, biphasic	29	17	88.2	5.9	0.0	5.9
	Thymoma	29	29	72.4	10.3	17.2	0.0
	Lung, neuroendocrine tumor (NET)	29	24	45.8	0.0	33.3	20.8
Tumors of the female genital tract	Squamous cell carcinoma of the vagina	30	26	38.5	23.1	23.1	15.4
	Squamous cell carcinoma of the vulva	107	89	50.6	29.2	12.4	7.9
	Squamous cell carcinoma of the cervix	88	75	38.7	13.3	18.7	29.3
	Adenocarcinoma of the cervix	23	23	0.0	0.0	4.3	95.7
	Endometrioid endometrial carcinoma	288	266	3.8	3.4	12.4	80.5
	Endometrial serous carcinoma	36	33	24.2	9.1	24.2	42.4
	Carcinosarcoma of the uterus	57	46	30.4	4.3	26.1	39.1
	Endometrial carcinoma, high grade, G3	13	13	53.8	7.7	15.4	23.1
	Endometrial clear cell carcinoma	9	8	37.5	0.0	0.0	62.5
	Endometrioid carcinoma of the ovary	93	71	9.9	8.5	15.5	66.2
	Serous carcinoma of the ovary	530	439	32.8	19.1	32.1	15.9
	Mucinous carcinoma of the ovary	75	51	0.0	0.0	0.0	100.0
	Clear cell carcinoma of the ovary	51	40	10.0	7.5	30.0	52.5
	Carcinosarcoma of the ovary	47	35	54.3	14.3	22.9	8.6
	Granulosa cell tumor of the ovary	44	42	95.2	2.4	0.0	2.4
	Leydig cell tumor of the ovary	4	4	75.0	25.0	0.0	0.0
	Sertoli cell tumor of the ovary	1	1	100.0	0.0	0.0	0.0
	Sertoli Leydig cell tumor of the ovary	3	3	100.0	0.0	0.0	0.0
	Steroid cell tumor of the ovary	3	3	66.7	33.3	0.0	0.0
	Brenner tumor	32	28	3.6	0.0	0.0	96.4
Tumors of the breast	Invasive breast carcinoma of no special type	499	344	10.2	4.4	5.5	79.9
	Lobular carcinoma of the breast	150	107	2.8	0.9	2.8	93.5
	Medullary carcinoma of the breast	8	7	42.9	14.3	0.0	42.9
	Tubular carcinoma of the breast	2	1	0.0	0.0	0.0	100.0
	Mucinous carcinoma of the breast	7	5	0.0	0.0	0.0	100.0
Tumors of the digestive system	Adenomatous polyp, low‐grade dysplasia	50	26	0.0	0.0	0.0	100.0
	Adenomatous polyp, high‐grade dysplasia	50	38	0.0	0.0	0.0	100.0
	Adenocarcinoma of the colon	2483	2236	0.4	0.7	1.4	97.5
	Gastric adenocarcinoma, diffuse type	215	191	1.6	0.0	0.5	97.9
	Gastric adenocarcinoma, intestinal type	215	186	5.4	1.1	4.8	88.7
	Gastric adenocarcinoma, mixed type	62	48	2.1	2.1	2.1	93.8
	Adenocarcinoma of the esophagus	83	78	6.4	1.3	5.1	87.2
	Squamous cell carcinoma of the esophagus	76	70	35.7	17.1	25.7	21.4
	Squamous cell carcinoma of the anal canal	91	65	43.1	20.0	9.2	27.7
	Cholangiocarcinoma	58	46	34.8	4.3	8.7	52.2
	Gallbladder adenocarcinoma	51	46	4.3	2.2	2.2	91.3
	Gallbladder Klatskin tumor	42	33	12.1	0.0	0.0	87.9
	Hepatocellular carcinoma	312	277	61.4	6.5	7.2	24.9
	Ductal adenocarcinoma of the pancreas	659	435	1.8	0.5	2.8	94.9
	Pancreatic/Ampullary adenocarcinoma	98	69	4.3	1.4	1.4	92.8
	Acinar cell carcinoma of the pancreas	18	17	23.5	5.9	5.9	64.7
	Gastrointestinal stromal tumor (GIST)	62	60	98.3	0.0	1.7	0.0
	Appendix, neuroendocrine tumor (NET)	25	16	25.0	0.0	25.0	50.0
	Colorectal, neuroendocrine tumor (NET)	12	9	11.1	0.0	11.1	77.8

	Ileum, neuroendocrine tumor (NET)	53	45	4.4	2.2	13.3	80.0
	Pancreas, neuroendocrine tumor (NET)	101	76	55.3	7.9	18.4	18.4
	Colorectal, neuroendocrine carcinoma (NEC)	14	12	50.0	0.0	0.0	50.0
	Ileum, neuroendocrine carcinoma (NEC)	8	7	0.0	0.0	0.0	100.0
	Gallbladder, neuroendocrine carcinoma (NEC)	4	4	75.0	0.0	0.0	25.0
Pancreas, neuroendocrine carcinoma (NEC)	14	9	44.4	11.1	33.3	11.1
Tumors of the urinary system	Noninvasive papillary urothelial carcinoma, pTa G2 low grade	87	81	0.0	0.0	0.0	100.0
	Noninvasive papillary urothelial carcinoma, pTa G2 high grade	80	76	0.0	0.0	1.3	98.7
	Noninvasive papillary urothelial carcinoma, pTa G3	126	117	0.0	3.4	6.0	90.6
	Urothelial carcinoma, pT2‐4 G3	735	540	19.1	11.7	13.3	55.9
	Squamous cell carcinoma of the bladder	22	21	42.9	38.1	9.5	9.5
	Small cell neuroendocrine carcinoma of the bladder	5	5	80.0	0.0	0.0	20.0
	Sarcomatoid urothelial carcinoma	25	12	50.0	16.7	25.0	8.3
	Urothelial carcinoma of the kidney pelvis	62	59	25.4	11.9	13.6	49.2
	Clear cell renal cell carcinoma	1287	1048	92.2	4.5	1.5	1.8
	Papillary renal cell carcinoma	368	275	73.5	16.4	6.2	4.0
	Clear cell (tubulo) papillary renal cell carcinoma	26	17	88.2	0.0	5.9	5.9
	Chromophobe renal cell carcinoma	170	132	84.1	11.4	3.0	1.5
	Oncocytoma of the kidney	257	190	63.2	21.6	13.7	1.6
Tumors of the male genital organs	Adenocarcinoma of the prostate, Gleason 3 + 3	83	83	3.6	3.6	8.4	84.3
	Adenocarcinoma of the prostate, Gleason 4 + 4	80	80	12.5	3.8	15.0	68.8
	Adenocarcinoma of the prostate, Gleason 5 + 5	85	85	14.1	4.7	20.0	61.2
	Adenocarcinoma of the prostate (recurrence)	258	240	11.3	6.3	18.8	63.8
	Small cell neuroendocrine carcinoma of the prostate	2	2	0.0	0.0	50.0	50.0
	Seminoma	682	649	99.1	0.2	0.8	0.0
	Embryonal carcinoma of the testis	54	45	93.3	0.0	6.7	0.0
	Leydig cell tumor of the testis	31	30	90.0	10.0	0.0	0.0
	Sertoli cell tumor of the testis	2	2	50.0	50.0	0.0	0.0
	Sex cord‐stromal tumor of the testis	1	1	100.0	0.0	0.0	0.0
	Spermatocytic tumor of the testis	1	1	100.0	0.0	0.0	0.0
	Yolk sac tumor	53	41	80.5	0.0	17.1	2.4
	Teratoma	53	41	65.9	2.4	19.5	12.2
	Squamous cell carcinoma of the penis	92	69	53.6	21.7	8.7	15.9
Tumors of endocrine organs	Adenoma of the thyroid gland	63	63	38.1	15.9	14.3	31.7
	Papillary thyroid carcinoma	341	330	10.3	7.9	10.6	71.2
	Follicular thyroid carcinoma	109	106	34.0	13.2	16.0	36.8
	Medullary thyroid carcinoma	57	56	3.6	7.1	3.6	85.7
	Parathyroid gland adenoma	43	41	87.8	4.9	7.3	0.0
	Anaplastic thyroid carcinoma	19	19	68.4	10.5	10.5	10.5
	Adrenal cortical adenoma	48	43	90.7	9.3	0.0	0.0
	Adrenal cortical carcinoma	27	23	95.7	4.3	0.0	0.0
	Pheochromocytoma	51	48	100.0	0.0	0.0	0.0
Tumors of hematopoietic and lymphoid tissues	Hodgkin's lymphoma	103	93	100.0	0.0	0.0	0.0
	Small lymphocytic lymphoma, B‐cell type (B‐SLL/B‐CLL)	50	39	100.0	0.0	0.0	0.0
	Diffuse large B‐cell lymphoma (DLBCL)	113	101	98.0	1.0	1.0	0.0
	Follicular lymphoma	88	77	100.0	0.0	0.0	0.0
	T‐cell non‐Hodgkin's lymphoma	25	18	100.0	0.0	0.0	0.0
	Mantle cell lymphoma	18	15	100.0	0.0	0.0	0.0
	Marginal zone lymphoma	16	12	100.0	0.0	0.0	0.0
	Diffuse large B‐cell lymphoma (DLBCL) in the testis	16	14	100.0	0.0	0.0	0.0
	Burkitt lymphoma	5	2	100.0	0.0	0.0	0.0
Tumors of soft tissue and bone	Granular cell tumor	23	13	100.0	0.0	0.0	0.0
	Leiomyoma	50	49	100.0	0.0	0.0	0.0
	Leiomyosarcoma	94	90	100.0	0.0	0.0	0.0
	Liposarcoma	96	81	100.0	0.0	0.0	0.0
	Malignant peripheral nerve sheath tumor (MPNST)	15	13	92.3	0.0	0.0	7.7
	Myofibrosarcoma	26	25	100.0	0.0	0.0	0.0
	Angiosarcoma	42	28	100.0	0.0	0.0	0.0
	Angiomyolipoma	91	69	89.9	10.1	0.0	0.0
	Dermatofibrosarcoma protuberans	21	11	100.0	0.0	0.0	0.0
	Ganglioneuroma	14	10	100.0	0.0	0.0	0.0
	Kaposi sarcoma	8	2	100.0	0.0	0.0	0.0
	Neurofibroma	117	82	100.0	0.0	0.0	0.0
	Sarcoma, not otherwise specified (NOS)	74	62	96.8	0.0	3.2	0.0

	Paraganglioma	41	28	96.4	3.6	0.0	0.0
	Ewing sarcoma	23	12	100.0	0.0	0.0	0.0
	Rhabdomyosarcoma	7	6	100.0	0.0	0.0	0.0
	Schwannoma	122	92	100.0	0.0	0.0	0.0
	Synovial sarcoma	12	9	100.0	0.0	0.0	0.0
	Osteosarcoma	19	11	90.9	9.1	0.0	0.0
	Chondrosarcoma	15	8	100.0	0.0	0.0	0.0
	Rhabdoid tumor	5	5	100.0	0.0	0.0	0.0
Solitary fibrous tumor	17	17	100.0	0.0	0.0	0.0

**TABLE 2:** AGR2 immunostaining and tumor phenotype.

		AGR2 immunostaining					
		*n*	Negative (%)	Weak (%)	Moderate (%)	Strong (%)	*p*
Colorectal cancers	All cancers	2168	0.5	0.7	1.3	97.5	
	pT1	83	1.2	0	1.2	97.6	0.1215
	pT2	421	0	0.2	0.7	99	
	pT3	1200	0.5	0.6	1.4	97.5	
	pT4	430	0.7	1.6	1.6	96	
	pN0	1109	0.2	0.5	0.9	98.4	0.0275
	pN+	1018	0.8	0.9	1.9	98.5	
	V0	1535	0.3	0.5	1.4	97.9	0.0514
	V+	558	1.1	1.3	1.3	96.4	
	L0	696	0.1	0.7	0.9	98.3	0.1817
	L1	1407	0.6	0.7	1.6	97.1	
	Left colon	1228	0.2	0.6	1.1	98	0.3608
	Right colon	465	0.9	0.4	1.5	97.2	
	MSI	88	0	0	1.1	98.9	0.7543
	MSS	1158	0.2	0.5	1.3	98	
	RAS mutated	356	0	0	1.4	98.6	0.0136
	RAS wildtype	451	0.7	1.3	1.1	96.9	
	BRAF mutated	22	0	0	0	100	0.6245
	BRAF wildtype	131	0	0.8	1.5	97.7	
Endometrioid endometrial carcinoma	pT1	104	1.9	2.9	12.5	82.7	0.297
	pT2	24	4.2	0	29.2	66.7	
	pT3‐4	37	5.4	5.4	18.9	70.3	
	pN0	50	4	0	20	76	0.0482
	pN+	30	0	10	16.7	73.3	
Serous carcinoma of the ovary	pT1	30	33.3	20	20	26.7	0.2592
	pT2	42	42.9	23.8	21.4	11.9	
	pT3	245	32.2	16.3	33.9	17.6	
	pN0	80	28.8	22.5	26.3	22.5	0.1028
	pN1	158	39.9	17.1	30.4	12.7	
Endometrioid carcinoma of the ovary	pT1	26	0	11.5	19.2	69.2	0.2769
	pT2	5	0	20	0	80	
	pT3	6	16.7	16.7	0	66.7	
	pN0	24	0	12.5	4.2	83.3	0.0673
	pN1	8	12.5	0	25	62.5	
Clear cell renal cell carcinoma	all cancers	1001	96.9	0.7	0.7	1.7	
	ISUP						
	1	227	99.6	0	0	0.4	0.0013
	2	356	97.8	0.3	0.3	1.7	
	3	219	95.9	0.5	0.9	2.7	
	4	70	87.1	5.7	1.4	5.7	
	Fuhrman						
	1	55	98.2	0	0	1.8	< 0.0001
	2	596	98.8	0.2	0.2	0.8	
	3	249	96.4	0.4	1.6	1.6	
	4	84	83.3	6	2.4	8.3	
	Thoenes						
	1	302	100	0	0	0	< 0.0001
	2	414	97.1	0.2	0.7	1.9	
	3	92	85.9	5.4	2.2	6.5	
	UICC						
	1	259	97.7	0.8	0	1.5	0.2976
	2	31	96.8	0	3.2	0	
	3	83	97.6	1.2	0	1.2	
	4	66	92.4	3	1.5	3	
	pT1	569	98.2	0.4	0.4	1.1	0.0035
	pT2	118	99.2	0	0.8	0	
	pT3‐4	303	93.4	1.7	1.3	3.6	
	pN0	149	96	2	0	2	0.5371
	pN+	25	96	0	0	4	
	pM0	88	98.9	0	0	1.1	0.0542
	pM+	86	91.9	3.5	2.3	2.3	
Papillary renal cell carcinoma	All cancers	240	72.1	17.9	6.3	3.8	
	ISUP						
	1	29	89.7	6.9	3.4	0	0.194
	2	112	64.3	21.4	8.9	5.4	
	3	64	71.9	21.9	3.1	3.1	
	4	5	80	20	0	0	
	Fuhrman						

	1	2	100	0	0	0	0.5608
	2	151	70.9	17.9	7.9	3.3	
	3	65	73.8	21.5	1.5	3.1	
	4	9	77.8	11.1	11.1	0	
	Thoenes						
	1	45	77.8	15.6	6.7	0	0.2377
	2	128	66.4	23.4	6.3	3.9	
	3	15	86.7	6.7	6.7	0	
	UICC						
	1	76	72.4	15.8	6.6	5.3	0.7206
	2	11	63.6	27.3	9.1	0	
	3	4	50	25	25	0	
	4	9	55.6	11.1	22.2	11.1	
	pT1	166	72.3	17.5	6.6	3.6	0.9371
	pT2	41	70.7	22	4.9	2.4	
	pT3‐4	27	70.4	18.5	3.7	7.4	
	pN0	18	50	27.8	22.2	0	0.1321
	pN+	13	61.5	7.7	15.4	15.4	
	pM0	22	40.9	27.3	18.2	13.6	0.3536
pM+	11	72.7	9.1	9.1	9.1		
Ductal adenocarcinoma of the pancreas	All cancers	327	2.1	0.6	3.1	94.2	
	pT1	7	0	0	0	100	0.8717
	pT2	44	2.3	2.3	2.3	93.2	
	pT3	259	1.9	0.4	3.5	94.2	
	pT4	17	5.9	0	0	94.1	
	G1	9	0	0	0	100	0.073
	G2	230	2.6	0.9	1.7	94.8	
	G3	70	0	0	8.6	91.4	
	pN0	65	0	1.5	3.1	95.4	0.3114
	pN+	261	2.3	0.4	3.1	94.3	
Papillary thyroid carcinoma	pT1	152	9.2	10.5	9.2	71.1	0.086
	pT2	79	15.2	1.3	11.4	72.2	
	pT3‐4	97	8.2	9.3	12.4	70.1	
	pN0	91	11	9.9	11	68.1	0.1302
	pN+	121	3.3	7.4	12.4	76.9	
Follicular thyroid carcinoma	pT1	15	33.3	13.3	13.3	40	0.9763
	pT2	51	33.3	13.7	13.7	39.2	
	pT3‐4	38	34.2	13.2	21.1	31.6	
	pN0	30	46.7	3.3	10	40	0.3741
	pN+	2	50	0	50	0	
Invasive breast carcinoma of no special type	pT1	115	7.8	0.9	6.1	85.2	0.6069
	pT2	147	9.5	4.8	4.8	81	
	pT3‐4	27	7.4	3.7	3.7	85.2	
	G1	8	0	0	0	100	0.0049
	G2	162	4.3	1.9	4.3	89.5	
	G3	122	15.6	4.9	7.4	72.1	
	pN0	147	7.5	2	6.1	84.4	0.7739
	pN+	117	7.7	4.3	6	8.2	
Urothelial carcinoma	All cancers	637	11.3	7.5	9.9	71.3	
	pTa G2 low	81	0	0	0	100	0.0003
	pTa high	76	0	0	1.3	98.7	
	pTa G3	86	0	4.7	5.8	89.5	
	pT2	101	16.8	7.9	9.9	65.3	0.2196
	pT3	190	17.4	12.1	17.4	53.2	
	pT4	93	22.6	14	15.1	48.4	
	G2	18	0	5.6	5.6	88.9	0.0066
	G3	366	19.4	11.7	15.3	53.6	
	pN0	212	18.9	11.8	13.7	55.7	0.8212
	pN+	146	17.1	12.3	17.1	53.4	

## 4 Discussion

**FIGURE 4:**
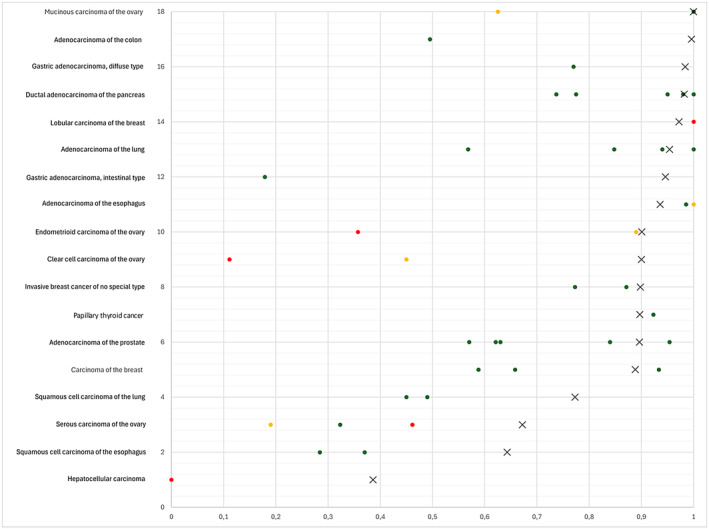
AGR2 protein expression in cancer (own findings vs. literature data). Graphical representation of AGR2 data from this study (X) compared to the previous literature. The colors of the dots represent the number of tumors analyzed in these studies: Red: *n* ≤ 20, yellow: *n* = 21 to 100, green: *n* > 100. For raw data and references, see Table S1.

The successful analysis of 12,434 tumors from 134 different tumor categories provides a comprehensive overview on AGR2 expression in cancer. The data show that the AGR2 findings in tumors largely mirror the observations in normal tissues. A significant AGR2 expression was found in large fractions of cases in virtually all epithelial tumor entities but only rarely in nonepithelial neoplasms. That adenocarcinomas are more commonly positive than squamous cell carcinomas reflects the limited AGR2 expression in normal squamous epithelium. That only few renal cell carcinomas were AGR2 positive also parallels our normal tissue findings as only few tubuli and collecting ducts had shown AGR2 staining in the normal kidney. For the few tumor entities that were previously analyzed for AGR2 expression, our positivity rates were generally in the upper range of earlier data (Figure 4). This demonstrates a comparatively high sensitivity of our assay. Accordingly, we found higher positivity rates for squamous cell carcinomas of the lung (77.3%) than the 49% of Fritzsche et al. [28] and the 45% of Pizzi et al. [24], for squamous cell carcinomas of the esophagus (64.3%) than the 28.4% of Takabatake et al. [29] and the 37% of DiMaio et al. [30] for clear cell carcinomas of the ovary (90%) than the 45% of Armes et al. [25] and the 11.1% of Darb‐Esfahani et al. [31] and for serous high‐grade carcinomas of the ovary (67.2%) than the 32.3% of Darb‐Esfahani et al. [31], the 50% of Park et al. [13], and the 19% of Armes et al. [25]. We also found an AGR2 positivity in 38.6% of our 277 hepatocellular carcinomas, a cancer type where Lepreux et al. [32] had previously not found AGR2 staining in a cohort of 12 cancers. Possible reasons that might have caused these discrepancies include differences in the staining protocols and antibodies used as well as different definitions of thresholds to determine positivity.

The comprehensive analysis of a broad range of cancer types and their corresponding normal tissues is an important prerequisite to assess the potential diagnostic utility of an IHC assay. The most striking diagnostic aspect for AGR2 was its significant upregulation in a large number of thyroidal neoplasms. While AGR2 expression was weak or absent in our normal thyroid, a moderate to strong AGR2 staining was observed in 46% of adenomas, 52.8% of follicular carcinomas, 81.8% of papillary carcinomas, and 31.6% of anaplastic carcinomas of the thyroid. AGR2 IHC may therefore be helpful for the distinction of neoplastic from nonneoplastic thyroidal tissues, which is especially challenging in cytology where sensitivity ranges from 45% to 95.2% [33, 34, 35, 36, 37, 38] depending on the subtype. Other potential diagnostic applications of AGR2 IHC may include the distinction of renal cell carcinomas (usually negative) from urothelial carcinomas (usually positive) from the kidney and mesotheliomas (usually negative) from adenocarcinomas (usually positive) of the lung. However, all these potential diagnostic applications need to be further evaluated.

The availability of large subsets of tumors from various important cancer entities enabled us to also address the clinical/prognostic relevance of AGR2 expression in diverse cancers. These data demonstrate that the prognostic role of AGR2 is cancer‐type dependent. In most cancers derived from tissues with high AGR2 expression in normal cells, such as the colorectum, the breast, the endometrium, and the urothelium, a reduced expression paralleled morphological parameters of tumor aggressiveness such as high histologic grade and advanced stage. These findings are in line with earlier data. Associations between reduced AGR2 expression and unfavorable tumor features have been described for colorectal cancer [39], ductal adenocarcinoma of the pancreas [40], prostate cancer [41], ovarian cancer [16, 25], and adenocarcinoma of the lung [42] although other groups could not confirm these findings for ovarian [31] and lung cancer [28] and others even found a link between low AGR expression and favorable tumor features for lung [10], ovarian, [31] and prostate cancer [23]. The reasons for the role of AGR2 loss in tumor progression are not clear. Functional studies have suggested a higher resistance to apoptosis [43] and a reduction of cellular adhesion [40, 43]. Alternatively, it is possible that reduced AGR2 expression in tumors derived from AGR2‐expressing normal cells just reflects tumor cell dedifferentiation which usually parallels cancer progression.

Elevated AGR2 expression was associated with unfavorable tumor features in only one of our cancer entities. The strong link of high AGR2 with high grade and advanced stage in clear cell renal cell carcinoma (ccRCC) is in line with RNA data from the Cancer Genome Atlas (TCGA) which also suggested a poor prognosis in case of high AGR2 expression levels [44]. Although the number of AGR2‐positive cases was low (7.8%) in this cancer entity, AGR2 expression analysis might become clinically useful in this cancer type because patients with high‐risk renal cell carcinomas are increasingly subjected to adjuvant chemotherapy, and risk assessment by histology alone is not sufficient for a safe individual risk assessment. Elevated levels of AGR2 expression have earlier also been linked to unfavorable tumor features in breast cancer [12], prostate cancer [23], head and neck squamous cell carcinoma [45], and squamous cell carcinoma of the esophagus [29].

Potential cancer promoting effects of AGR2 upregulation include a stimulating role on cell proliferation [29, 46, 47], migration [47, 48], epithelial–mesenchymal transition [48], invasion [49, 50], and chemoresistance [47]. Arumugam et al. [47] reported that AGR2 stimulated the proliferation, migration, invasion, and chemoresistance of pancreatic ductal adenocarcinoma cells by interacting with the metastasis‐associated protein C4.4A that is co‐expressed on the cell surface. Jia et al. [51] found that AGR2 directly interacts with VEGFA and enhances VEGFR/VEGFR2 signaling in prostate cancer cells, leading to the acquisition of a mesenchymal phenotype in vivo and in vitro, which is associated with increased invasiveness and metastatic growths. Fessart et al. [46] described a mechanism by which secreted AGR2 acts like a growth factor and stimulates cell proliferation in lung cancer cells by repressing cyclin‐dependent kinase inhibitor 1A (CDKN1A). Zhang et al. [48] reported that intracellular but not extracellular AGR2 promotes the expression of the SNAIL and SLUG transcription factors through direct transcriptional activation and histone 3 acetylation. This drives epithelial–mesenchymal transition in colorectal cancer cells [48], an important cellular process leading to increased migratory capability and invasive potential [52]. Others have found that extracellular AGR2 may promote cell migration and metastasis in CRC through noncanonical Wnt signaling [53]. Takabatake et al. [29] described a direct interaction of AGR2 with the p53 tumor suppressor that results in attenuation of p53 activity and increased cell proliferation in esophageal squamous cell carcinoma cells. Again, it cannot be excluded that AGR2 neo‐expression in tumors derived from non‐AGR2‐expressing normal cells is caused by random alterations occurring because of tumor cell dedifferentiation.

Additional interest in AGR2 is based on its potential as a therapeutic target. Wu et al. [54] showed inhibition of breast cancer growth in vitro using the first developed monoclonal antibody directed against AGR2, termed 184A. Subsequent studies showed the potential therapeutic benefit of a humanized version of 184A in ovarian cancer xenografts [55] and improved survival in lung cancer in mice [56]. Further therapeutic strategies are targeting the influence of AGR2 on chemotherapy resistance. Studies have shown that AGR2 is involved in cellular survival and the development of tamoxifen resistance in breast cancer [57]. Antibodies directed against AGR2 were able to reduce tumor growth in endocrine therapy‐resistant breast cancer [58].

Considering the large scale of our study, our assay was extensively validated according to the recommendations of the international working group of antibody validation (IWGAV) [59] by comparing our IHC findings in normal tissues with data obtained by another independent anti‐AGR2 antibody and RNA data derived from three different publicly accessible databases [60, 61, 62, 63]. To ensure an as broad as possible range of proteins to be tested for a possible cross‐reactivity, 76 different normal tissue categories were included in this analysis. These diverse tissues are likely to contain a large fraction of the proteins expressed in cells of adult humans all of which are screened for potential cross‐reactivities of antibodies. Our normal tissue analysis revealed AGR2 immunostaining in all organs for which RNA expression had been described (salivary glands, stomach, duodenum small intestine, appendix, colorectum, gallbladder, urinary bladder, lung, pituitary gland, epididymis, prostate, seminal vesicle, breast, cervix, and fallopian tube). While significant RNA expression had not been reported for other tissues with IHC‐positive cell types, such as endometrium, intercalated ducts and acinar cells of the pancreas, a subset of tubuli/collecting ducts of the kidney, squamous epithelial cells of the tonsil, hair follicles, and of corpuscles of Hassall's of the thymus, these staining obtained by our assay were confirmed by an independent second AGR2 antibody. It is likely that these cell types are made up of too small fractions of their entire organs to be detected in RNA analysis of disintegrated tissues. Overall, these validation data document a high level of specificity for our AGR2 IHC assay.

In summary, our data provide a comprehensive overview of AGR2 expression in different tumor entities, identify AGR2 IHC as a potential diagnostic aid for the identification of thyroidal neoplasms, and demonstrate a potential prognostic role of AGR2 in various cancer types.

## Author Contributions

**Nina Schraps:** conceptualization (equal), data curation (equal), formal analysis (equal), investigation (equal), methodology (equal), visualization (equal), writing – original draft (equal). **Jacob Constantin Port:** conceptualization (equal), data curation (equal), formal analysis (equal), investigation (equal), methodology (equal), visualization (equal), writing – original draft (equal). **Anne Menz:** data curation (equal), formal analysis (equal), investigation (equal), methodology (equal), resources (equal), writing – review and editing (supporting). **Florian Viehweger:** data curation (equal), formal analysis (equal), investigation (equal), methodology (equal), resources (equal), writing – review and editing (supporting). **Seyma Büyücek:** data curation (equal), formal analysis (equal), investigation (equal), methodology (equal), resources (equal), visualization (equal), writing – review and editing (supporting). **David Dum:** data curation (equal), formal analysis (equal), investigation (equal), methodology (equal), resources (equal), writing – review and editing (supporting). **Ria Schlichter:** data curation (equal), formal analysis (equal), investigation (equal), methodology (equal), resources (equal), writing – review and editing (supporting). **Andrea Hinsch:** data curation (equal), formal analysis (equal), investigation (equal), methodology (equal), resources (equal), writing – review and editing (supporting). **Christoph Fraune:** data curation (equal), formal analysis (equal), investigation (equal), methodology (equal), resources (equal), writing – review and editing (supporting). **Christian Bernreuther:** data curation (equal), formal analysis (equal), investigation (equal), methodology (equal), resources (equal), writing – review and editing (supporting). **Martina Kluth:** data curation (equal), formal analysis (equal), investigation (equal), methodology (equal), resources (equal), writing – review and editing (supporting). **Claudia Hube‐Magg:** data curation (equal), formal analysis (equal), investigation (equal), methodology (equal), resources (equal), writing – review and editing (supporting). **Katharina Möller:** data curation (equal), formal analysis (equal), investigation (equal), methodology (equal), resources (equal), writing – review and editing (supporting). **Viktor Reiswich:** data curation (equal), formal analysis (equal), investigation (equal), methodology (equal), resources (equal), writing – review and editing (supporting). **Andreas M. Luebke:** data curation (equal), formal analysis (equal), investigation (equal), methodology (equal), resources (equal), writing – review and editing (supporting). **Patrick Lebok:** data curation (equal), formal analysis (equal), investigation (equal), methodology (equal), resources (equal), writing – review and editing (supporting). **Sören Weidemann:** data curation (equal), formal analysis (equal), investigation (equal), methodology (equal), resources (equal), writing – review and editing (supporting). **Guido Sauter:** conceptualization (equal), data curation (equal), formal analysis (equal), investigation (equal), methodology (equal), resources (equal), supervision (equal), validation (equal), writing – original draft (equal), writing – review and editing (equal). **Maximilian Lennartz:** data curation (equal), formal analysis (equal), investigation (equal), methodology (equal), resources (equal), writing – review and editing (supporting). **Frank Jacobsen:** data curation (equal), formal analysis (equal), investigation (equal), methodology (equal), resources (equal), writing – review and editing (supporting). **Till S. Clauditz:** data curation (equal), formal analysis (equal), investigation (equal), methodology (equal), resources (equal), writing – review and editing (supporting). **Andreas H. Marx:** data curation (equal), formal analysis (equal), investigation (equal), methodology (equal), resources (equal), writing – review and editing (supporting). **Ronald Simon:** conceptualization (equal), data curation (equal), formal analysis (equal), investigation (equal), methodology (equal), resources (equal), supervision (equal), validation (equal), writing – original draft (equal), writing – review and editing (equal). **Stefan Steurer:** data curation (equal), formal analysis (equal), investigation (equal), methodology (equal), resources (equal), writing – review and editing (supporting). **Baris Mercanoglu:** data curation (equal), formal analysis (equal), investigation (equal), methodology (equal), resources (equal), writing – review and editing (supporting). **Nathaniel Melling:** data curation (equal), formal analysis (equal), investigation (equal), methodology (equal), resources (equal), writing – review and editing (supporting). **Thilo Hackert:** data curation (equal), formal analysis (equal), investigation (equal), methodology (equal), resources (equal), writing – review and editing (equal). **Eike Burandt:** data curation (equal), formal analysis (equal), investigation (equal), methodology (equal), resources (equal), writing – review and editing (supporting). **Natalia Gorbokon:** data curation (equal), formal analysis (equal), investigation (equal), methodology (equal), resources (equal), writing – review and editing (supporting). **Sarah Minner:** data curation (equal), formal analysis (equal), investigation (equal), methodology (equal), resources (equal), writing – review and editing (supporting). **Till Krech:** data curation (equal), formal analysis (equal), investigation (equal), methodology (equal), resources (equal), writing – review and editing (supporting). **Florian Lutz:** conceptualization (equal), data curation (equal), formal analysis (equal), investigation (equal), methodology (equal), resources (equal), supervision (equal), writing – original draft (equal), writing – review and editing (equal).

## Ethics Statement

The use of archived remnants of diagnostic tissues for manufacturing of TMAs and their analysis for research purposes as well as patient data analysis has been approved by local laws (HmbKHG, §12) and by the local ethics committee (Ethics commission Hamburg, WF‐049/09), allowing the use of leftover diagnostic tissues and corresponding clinical data for scientific research without the patients written consent. All work has been carried out in compliance with the Helsinki Declaration.

## Conflicts of Interest

The AGR2 antibody clone HMV‐325 was provided by ardoci GmbH (owned by a family member of G.S.).

## Supporting information


Data S1.

Data S2.

## Data Availability

All data generated or analyzed during this study are included in this published article.

## References

[1] D. Jach , Y. Cheng , F. Prica , L. Dumartin , and T. Crnogorac‐Jurcevic , “From Development to Cancer ‐ An Ever‐Increasing Role of AGR2,” American Journal of Cancer Research 11 (2021): 5249–5262. 34873459 PMC8640830

[2] F. Aberger , G. Weidinger , H. Grunz , and K. Richter , “Anterior Specification of Embryonic Ectoderm: The Role of the Xenopus Cement Gland‐Specific Gene XAG‐2,” Mechanisms of Development 72 (1998): 115–130. 9533957 10.1016/s0925-4773(98)00021-5

[3] Q. Zhu , H. B. Mangukiya , D. S. Mashausi , et al., “Anterior Gradient 2 Is Induced in Cutaneous Wound and Promotes Wound Healing Through Its Adhesion Domain,” FEBS Journal 284 (2017): 2856–2869. 28665039 10.1111/febs.14155

[4] S.‐W. Park , G. Zhen , C. Verhaeghe , et al., “The Protein Disulfide Isomerase AGR2 Is Essential for Production of Intestinal Mucus,” Proceedings of the National Academy of Sciences of the United States of America 106 (2009): 6950–6955. 19359471 10.1073/pnas.0808722106 PMC2678445

[5] M. B. Tereshina , A. S. Ivanova , F. M. Eroshkin , et al., “Agr2‐Interacting Prod1‐Like Protein Tfp4 From *Xenopus laevis* Is Necessary for Early Forebrain and Eye Development as Well as for the Tadpole Appendage Regeneration,” Genes N. Y. N 2000 57 (2019): e23293. 10.1002/dvg.23293 30912273

[6] A. S. Ivanova , M. B. Tereshina , G. V. Ermakova , V. V. Belousov , and A. G. Zaraisky , “Agr Genes, Missing in Amniotes, Are Involved in the Body Appendages Regeneration in Frog Tadpoles,” Scientific Reports 3 (2013): 1279. 23412115 10.1038/srep01279 PMC3573343

[7] A. A. Al‐Shaibi , “Human AGR2 Deficiency Causes Mucus Barrier Dysfunction and Infantile Inflammatory Bowel Disease,” Cellular and Molecular Gastroenterology and Hepatology 12 (2021): 1809–1830. 34237462 10.1016/j.jcmgh.2021.07.001 PMC8551217

[8] F. Zhao , R. Edwards , D. Dizon , et al., “Disruption of Paneth and Goblet Cell Homeostasis and Increased Endoplasmic Reticulum Stress in Agr2−/− Mice,” Developmental Biology 338 (2010): 270–279. 20025862 10.1016/j.ydbio.2009.12.008 PMC2937056

[9] L. Dumartin , H. J. Whiteman , M. E. Weeks , et al., “AGR2 Is a Novel Surface Antigen That Promotes the Dissemination of Pancreatic Cancer Cells Through Regulation of Cathepsins B and *D* ,” Cancer Research 71 (2011): 7091–7102. 21948970 10.1158/0008-5472.CAN-11-1367 PMC3541941

[10] H. Ci and L. Wu , “Expression of KAI1 and AGR2 in Lung Adenocarcinoma and Their Clinicopathological Significance,” Medicine (Baltimore) 101 (2022): e32498. 36595821 10.1097/MD.0000000000032498 PMC9794224

[11] G. Zhang , X. Wang , C. Li , et al., “Integrated Stress Response Couples Mitochondrial Protein Translation With Oxidative Stress Control,” Circulation 144 (2021): 1500–1515. 34583519 10.1161/CIRCULATIONAHA.120.053125 PMC8563444

[12] D. L. Barraclough , A. Platt‐Higgins , S. de Silva Rudland , et al., “The Metastasis‐Associated Anterior Gradient 2 Protein Is Correlated With Poor Survival of Breast Cancer Patients,” American Journal of Pathology 175 (2009): 1848–1857. 19834055 10.2353/ajpath.2009.090246 PMC2774050

[13] K. Park , Y. J. Chung , H. So , et al., “AGR2, a Mucinous Ovarian Cancer Marker, Promotes Cell Proliferation and Migration,” Experimental & Molecular Medicine 43 (2011): 91–100. 21200134 10.3858/emm.2011.43.2.011 PMC3047197

[14] R. Hrstka , P. Bouchalova , E. Michalova , et al., “AGR2 Oncoprotein Inhibits p38 MAPK and p53 Activation Through a DUSP10‐Mediated Regulatory Pathway,” Molecular Oncology 10 (2016): 652–662. 26733232 10.1016/j.molonc.2015.12.003 PMC5423154

[15] Z. Wang , Y. Hao , and A. W. Lowe , “The Adenocarcinoma‐Associated Antigen, AGR2, Promotes Tumor Growth, Cell Migration, and Cellular Transformation,” Cancer Research 68 (2008): 492–497. 18199544 10.1158/0008-5472.CAN-07-2930

[16] M. R. Alves , N. C. e Melo , M. C. Barros‐Filho , et al., “Downregulation of agr 2, p21, and Cyclin D and Alterations in p53 Function Were Associated With Tumor Progression and Chemotherapy Resistance in Epithelial Ovarian Carcinoma,” Cancer Medicine 7 (2018): 3188–3199. 29845750 10.1002/cam4.1530 PMC6051166

[17] F. R. Fritzsche , E. Dahl , S. Pahl , et al., “Prognostic Relevance of AGR2 Expression in Breast Cancer,” Clinical Cancer Research: An Official Journal of the American Association for Cancer Research 12 (2006): 1728–1734. 16551856 10.1158/1078-0432.CCR-05-2057

[18] J. Guo , G. Gong , and B. Zhang , “Identification and Prognostic Value of Anterior Gradient Protein 2 Expression in Breast Cancer Based on Tissue Microarray,” Tumour Biology: The Journal of the International Society for Oncodevelopmental Biology and Medicine 39 (2017): 1010428317713392. 28671019 10.1177/1010428317713392

[19] M. Zhou , X. L. Gan , Y. X. Ren , et al., “AGR2 and FOXA1 as Prognostic Markers in ER‐Positive Breast Cancer,” BMC Cancer 23 (2023): 743. 37568077 10.1186/s12885-023-10964-6 PMC10416444

[20] H. Bu , M. R. Schweiger , T. Manke , et al., “Anterior Gradient 2 and 3– Two Prototype Androgen‐Responsive Genes Transcriptionally Upregulated by Androgens and by Oestrogens in Prostate Cancer Cells,” FEBS Journal 280 (2013): 1249–1266. 23294566 10.1111/febs.12118

[21] H. Bu , S. Bormann , G. Schäfer , et al., “The Anterior Gradient 2 (AGR2) Gene Is Overexpressed in Prostate Cancer and May Be Useful as a Urine Sediment Marker for Prostate Cancer Detection,” Prostate 71 (2011): 575–587. 20945500 10.1002/pros.21273

[22] J. Zhang , Y. Jin , S. Xu , et al., “AGR2 Is Associated With Gastric Cancer Progression and Poor Survival,” Oncology Letters 11 (2016): 2075–2083. 26998125 10.3892/ol.2016.4160 PMC4774612

[23] Y. Zhang , S. S. Forootan , D. Liu , et al., “Increased Expression of Anterior Gradient‐2 Is Significantly Associated With Poor Survival of Prostate Cancer Patients,” Prostate Cancer and Prostatic Diseases 10 (2007): 293–300. 17457305 10.1038/sj.pcan.4500960

[24] M. Pizzi , M. Fassan , M. Balistreri , A. Galligioni , F. Rea , and M. Rugge , “Anterior Gradient 2 Overexpression in Lung Adenocarcinoma,” Applied Immunohistochemistry & Molecular Morphology: AIMM 20 (2012): 31–36. 21768879 10.1097/PAI.0b013e3182233f9f

[25] J. E. Armes , C. M. Davies , S. Wallace , T. Taheri , L. C. Perrin , and D. J. Autelitano , “AGR2 Expression in Ovarian Tumours: A Potential Biomarker for Endometrioid and Mucinous Differentiation,” Pathology (Philadelphia, Pa.) 45 (2013): 49–54. 10.1097/PAT.0b013e32835bd561 23222243

[26] A.‐M. Dancau , R. Simon , M. Mirlacher , and G. Sauter , “Tissue Microarrays,” Methods in Molecular Biology (Clifton, N.J.) 1381 (2016): 53–65. 10.1007/978-1-4939-3204-7_3 26667454

[27] J. Kononen , L. Bubendorf , A. Kallionimeni , et al., “Tissue Microarrays for High‐Throughput Molecular Profiling of Tumor Specimens,” Nature Medicine 4 (1998): 844–847. 10.1038/nm0798-844 9662379

[28] F. R. Fritzsche , E. Dahl , A. Dankof , et al., “Expression of AGR2 in Non Small Cell Lung Cancer,” Histology and Histopathology 22 (2007): 703–708. 17455144 10.14670/HH-22.703

[29] K. Takabatake , H. Konishi , T. Arita , et al., “Anterior Gradient 2 Regulates Cancer Progression in TP53‐Wild‐Type Esophageal Squamous Cell Carcinoma,” Oncology Reports 46 (2021): 260. 34713298 10.3892/or.2021.8211

[30] M. A. DiMaio , S. Kwok , K. D. Montgomery , A. W. Lowe , and R. K. Pai , “Immunohistochemical Panel for Distinguishing Esophageal Adenocarcinoma From Squamous Cell Carcinoma: A Combination of p63, Cytokeratin 5/6, MUC5AC, and Anterior Gradient Homolog 2 Allows Optimal Subtyping,” Human Pathology 43 (2012): 1799–1807. 22748473 10.1016/j.humpath.2012.03.019 PMC3465493

[31] S. Darb‐Esfahani , F. Fritzsche , G. Kristiansen , et al., “Anterior Gradient Protein 2 (AGR2) is an Independent Prognostic Factor in Ovarian High‐Grade Serous Carcinoma,” Virchows Archiv: An International Journal of Pathology 461 (2012): 109–116. 22752467 10.1007/s00428-012-1273-4

[32] S. Lepreux , P. Bioulac‐Sage , and E. Chevet , “Differential Expression of the Anterior Gradient Protein‐2 Is a Conserved Feature During Morphogenesis and Carcinogenesis of the Biliary Tree,” Liver International: Official Journal of the International Association for the Study of the Liver 31 (2011): 322–328. 21281432 10.1111/j.1478-3231.2010.02438.x

[33] P. Trimboli , G. Treglia , L. Guidobaldi , et al., “Detection Rate of FNA Cytology in Medullary Thyroid Carcinoma: A Meta‐Analysis,” Clinical Endocrinology 82 (2015): 280–285. 25047365 10.1111/cen.12563

[34] F. Hajmanoochehri and E. Rabiee , “FNAC Accuracy in Diagnosis of Thyroid Neoplasms Considering all Diagnostic Categories of the Bethesda Reporting System: A Single‐Institute Experience,” Journal of Cytology 32 (2015): 238–243. 26811571 10.4103/0970-9371.171234 PMC4707785

[35] E. A. Sinna and N. Ezzat , “Diagnostic Accuracy of Fine Needle Aspiration Cytology in Thyroid Lesions,” Journal of the Egyptian National Cancer Institute 24 (2012): 63–70. 23582597 10.1016/j.jnci.2012.01.001

[36] M. A. Musani , F. A. Khan , S. Malik , and Y. Khambaty , “Fine Needle Aspiration Cytology: Sensitivity and Specificity in Thyroid Lesions,” Journal of Ayub Medical College, Abbottabad: JAMC 23 (2011): 34–36. 22830141

[37] R. Elisei , V. Bottici , F. Luchetti , et al., “Impact of Routine Measurement of Serum Calcitonin on the Diagnosis and Outcome of Medullary Thyroid Cancer: Experience in 10,864 Patients With Nodular Thyroid Disorders,” Journal of Clinical Endocrinology and Metabolism 89 (2004): 163–168. 14715844 10.1210/jc.2003-030550

[38] J. Cáp , A. Ryska , P. Rehorková , E. Hovorková , Z. Kerekes , and D. Pohnetalová , “Sensitivity and Specificity of the Fine Needle Aspiration Biopsy of the Thyroid: Clinical Point of View,” Clinical Endocrinology 51 (1999): 509–515. 10583320 10.1046/j.1365-2265.1999.00847.x

[39] M.‐O. Riener , T. Thiesler , C. Hellerbrand , et al., “Loss of Anterior Gradient‐2 Expression Is an Independent Prognostic Factor in Colorectal Carcinomas,” European Journal of Cancer (Oxford, England: 1990) 1990, no. 50 (2014): 1722–1730. 10.1016/j.ejca.2014.04.012 24794000

[40] Y. Mizuuchi , S. Aishima , K. Ohuchida , et al., “Anterior Gradient 2 Downregulation in a Subset of Pancreatic Ductal Adenocarcinoma Is a Prognostic Factor Indicative of Epithelial‐Mesenchymal Transition,” Laboratory Investigation; A Journal of Technical Methods and Pathology 95 (2015): 193–206. 25418581 10.1038/labinvest.2014.138

[41] M. E. Ho , S. I. Quek , L. D. True , et al., “Prostate Cancer Cell Phenotypes Based on AGR2 and CD10 Expression,” Modern Pathology: An Official Journal of the United States and Canadian Academy of Pathology, Inc 26 (2013): 849–859. 23348903 10.1038/modpathol.2012.238 PMC3638070

[42] K. Chung , N. Nishiyama , H. Wanibuchi , et al., “AGR2 as a Potential Biomarker of Human Lung Adenocarcinoma,” Osaka City Medical Journal 58 (2012): 13–24. 23094510

[43] D. Chanda , J. H. Lee , A. Sawant , et al., “Anterior Gradient Protein‐2 Is a Regulator of Cellular Adhesion in Prostate Cancer,” PLoS One 9 (2014): e89940. 24587138 10.1371/journal.pone.0089940 PMC3937391

[44] TCGA , “Research Network,” https://www.cancer.gov/tcga, 2024.

[45] B. Sun , Z. Cheng , and J. Sun , “Associations of MACC1, AGR2, and KAI1 Expression With the Metastasis and Prognosis in Head and Neck Squamous Cell Carcinoma,” International Journal of Clinical and Experimental Pathology 11 (2018): 822–830. 31938171 PMC6958041

[46] D. Fessart , C. de Barbeyrac , I. Boutin , et al., “Extracellular AGR2 Triggers Lung Tumour Cell Proliferation Through Repression of p21^CIP1^ ,” Biochimica et Biophysica Acta, Molecular Cell Research 1868 (2021): 118920. 33278424 10.1016/j.bbamcr.2020.118920

[47] T. Arumugam , D. Deng , L. Bover , H. Wang , C. D. Logsdon , and V. Ramachandran , “New Blocking Antibodies Against Novel AGR2‐C4.4A Pathway Reduce Growth and Metastasis of Pancreatic Tumors and Increase Survival in Mice,” Molecular Cancer Therapeutics 14 (2015): 941–951. 25646014 10.1158/1535-7163.MCT-14-0470 PMC4710371

[48] H. Zhang , J. Chi , J. Hu , et al., “Intracellular AGR2 Transduces PGE2 Stimuli to Promote Epithelial‐Mesenchymal Transition and Metastasis of Colorectal Cancer,” Cancer Letters 518 (2021): 180–195. 34216690 10.1016/j.canlet.2021.06.025

[49] T.‐T. Luu , D. H. Bach , D. Kim , R. Hu , H. J. Park , and S. K. Lee , “Overexpression of AGR2 Is Associated With Drug Resistance in Mutant Non‐small Cell Lung Cancers,” Anticancer Research 40 (2020): 1855–1866. 32234873 10.21873/anticanres.14139

[50] G. Di Maro , P. Salerno , K. Unger , et al., “Anterior Gradient Protein 2 Promotes Survival, Migration and Invasion of Papillary Thyroid Carcinoma Cells,” Molecular Cancer 13 (2014): 160. 24976026 10.1186/1476-4598-13-160 PMC4094684

[51] M. Jia , Y. Guo , D. Zhu , et al., “Pro‐Metastatic Activity of AGR2 Interrupts Angiogenesis Target Bevacizumab Efficiency via Direct Interaction With VEGFA and Activation of NF‐κB Pathway,” Biochimica et Biophysica Acta ‐ Molecular Basis of Disease 1864 (2018): 1622–1633. 29410027 10.1016/j.bbadis.2018.01.021

[52] R. Kalluri and R. A. Weinberg , “The Basics of Epithelial‐Mesenchymal Transition,” Journal of Clinical Investigation 119 (2009): 1420–1428. 19487818 10.1172/JCI39104 PMC2689101

[53] S. Tian , J. Hu , K. Tao , et al., “Secreted AGR2 Promotes Invasion of Colorectal Cancer Cells via Wnt11‐Mediated Non‐Canonical Wnt Signaling,” Experimental Cell Research 364 (2018): 198–207. 29427622 10.1016/j.yexcr.2018.02.004

[54] Z.‐H. Wu , Q. Zhu , G.‐W. Gao , C.‐C. Zhou , and D.‐W. Li , “Preparation, Characterization and Potential Application of Monoclonal Antibody 18A4 Against AGR2,” Xi Bao Yu Fen Zi Mian Yi Xue Za Zhi Chin Journal of Cellular and Molecular Immunology 26 (2010): 49–51. 20056089

[55] H. Guo , H. Chen , Q. Zhu , et al., “A Humanized Monoclonal Antibody Targeting Secreted Anterior Gradient 2 Effectively Inhibits the Xenograft Tumor Growth,” Biochemical and Biophysical Research Communications 475 (2016): 57–63. 27166154 10.1016/j.bbrc.2016.05.033

[56] H. Negi , S. B. Merugu , H. B. Mangukiya , et al., “Anterior Gradient‐2 Monoclonal Antibody Inhibits Lung Cancer Growth and Metastasis by Upregulating p53 Pathway and Without Exerting any Toxicological Effects: A Preclinical Study,” Cancer Letters 449 (2019): 125–134. 30685412 10.1016/j.canlet.2019.01.025

[57] R. Hrstka , R. Nenutil , A. Fourtouna , et al., “The Pro‐Metastatic Protein Anterior Gradient‐2 Predicts Poor Prognosis in Tamoxifen‐Treated Breast Cancers,” Oncogene 29 (2010): 4838–4847. 20531310 10.1038/onc.2010.228

[58] K. J. Cocce , J. S. Jasper , T. K. Desautels , et al., “The Lineage Determining Factor GRHL2 Collaborates With FOXA1 to Establish a Targetable Pathway in Endocrine Therapy‐Resistant Breast Cancer,” Cell Reports 29 (2019): 889–903.e10. 31644911 10.1016/j.celrep.2019.09.032 PMC6874102

[59] M. Uhlen , A. Bandrowski , S. Carr , et al., “A Proposal for Validation of Antibodies,” Nature Methods 13 (2016): 823–827. 27595404 10.1038/nmeth.3995 PMC10335836

[60] P. J. Thul , L. Åkesson , M. Wiking , et al., “A Subcellular Map of the Human Proteome,” Science 356 (2017): eaal3321. 28495876 10.1126/science.aal3321

[61] M. Lizio , J. Harshbarger , H. Shimoji , et al., “Gateways to the FANTOM5 Promoter Level Mammalian Expression Atlas,” Genome Biology 16 (2015): 22. 25723102 10.1186/s13059-014-0560-6 PMC4310165

[62] GTEx Consortium , “The Genotype‐Tissue Expression (GTEx) Project,” Nature Genetics 45 (2013): 580–585. 23715323 10.1038/ng.2653 PMC4010069

[63] M. Lizio , I. Abugessaisa , S. Noguchi , et al., “Update of the FANTOM Web Resource: Expansion to Provide Additional Transcriptome Atlases,” Nucleic Acids Research 47 (2019): D752–D758. 30407557 10.1093/nar/gky1099 PMC6323950

