# Supplementary material for: Prevalence and Significance of AGR2 Expression in Human Cancer
Source: Cancer Med. 2024 Nov 12;13(21):e70407. doi: 10.1002/cam4.70407 (PMC11557986; doi:10.1002/cam4.70407)
Supplement: Supplementary file 1 — Data S1. [file CAM4-13-e70407-s002.docx]

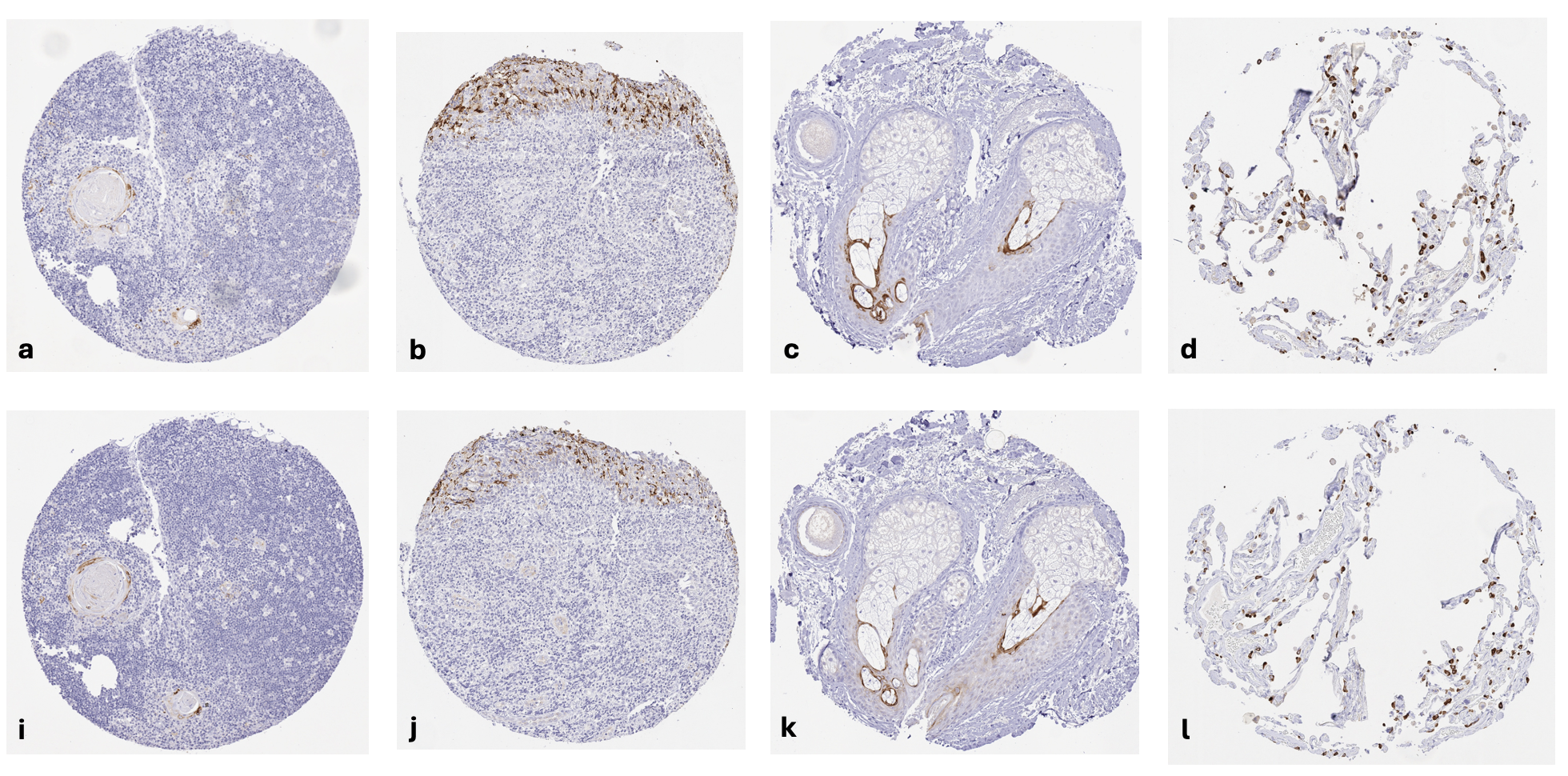


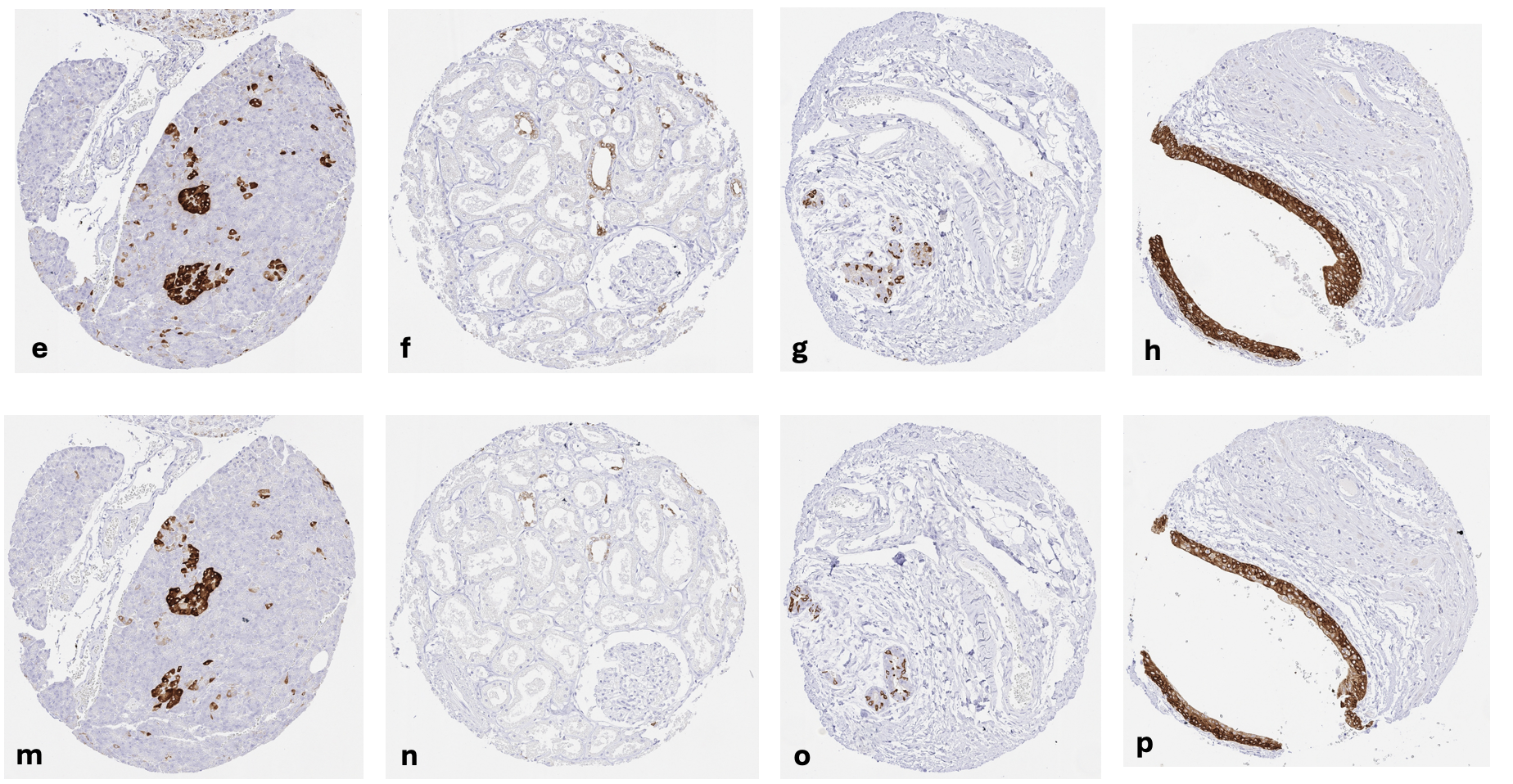


**Suppl Fig. 1.**

**IHC validation by comparison of two antibodies.** The panels show a concordance of immunostaining results obtained by two independent AGR2 antibodies. Using HMV-325, a predominantly cytoplasmic staining is seen in a subset of squamous epithelial cells in corpuscles of Hassall‘s of the thymus (**a**), a fraction of squamous epithelial cells of the tonsil (**b**), the inner (Huxley) layers of hair follicles of the skin (**c**), a large subset of pneumocytes of the lung (**d**), intercalated ducts and acinar cells of the pancreas (**e**), a small subset of tubuli/collecting ducts of the kidney (**f**), a subset of luminal cells in the breast (**g**), and of urothelial cells of the renal pelvis (**h**). Using clone EPR3278, a comparable staining was seen in the thymus (**i**), tonsil (**j**), hair follicles of the skin (**k**), lung (**l**), pancreas (**m**), kidney (**n**), breast (**o**), and the urothelium (**p**). The images a-h and i-p are from consecutive tissue sections.

**Suppl Tab. 1.**

**List of raw data and references used to create Figure 5.**

**
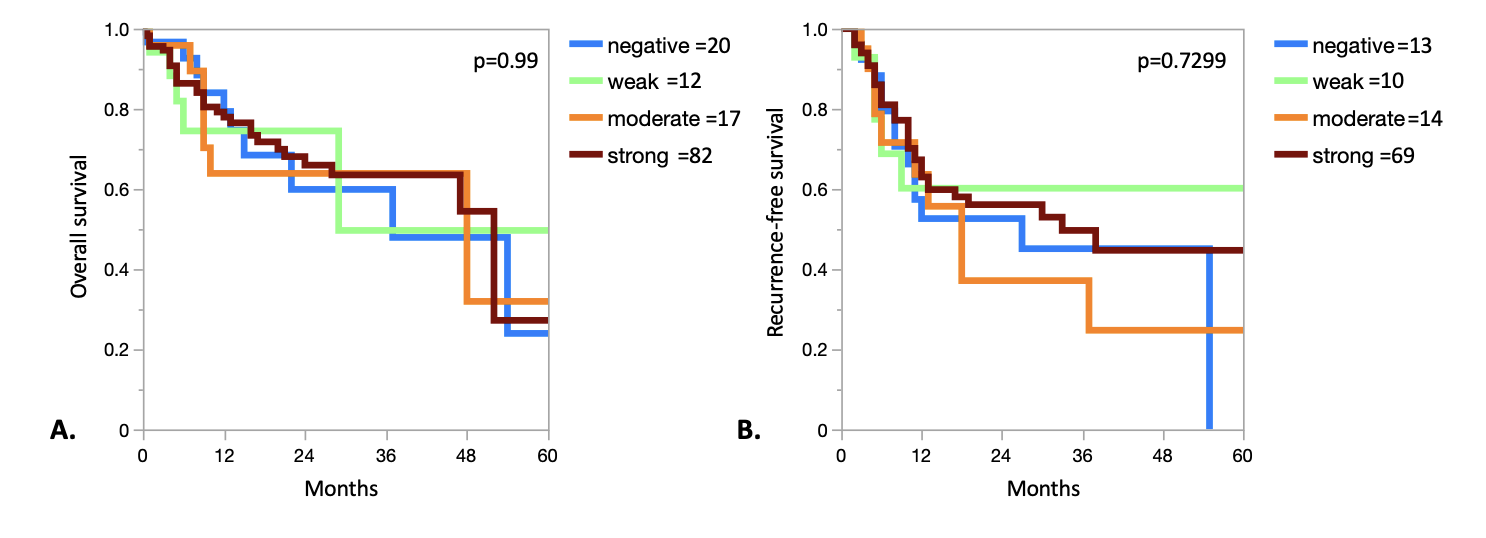
**


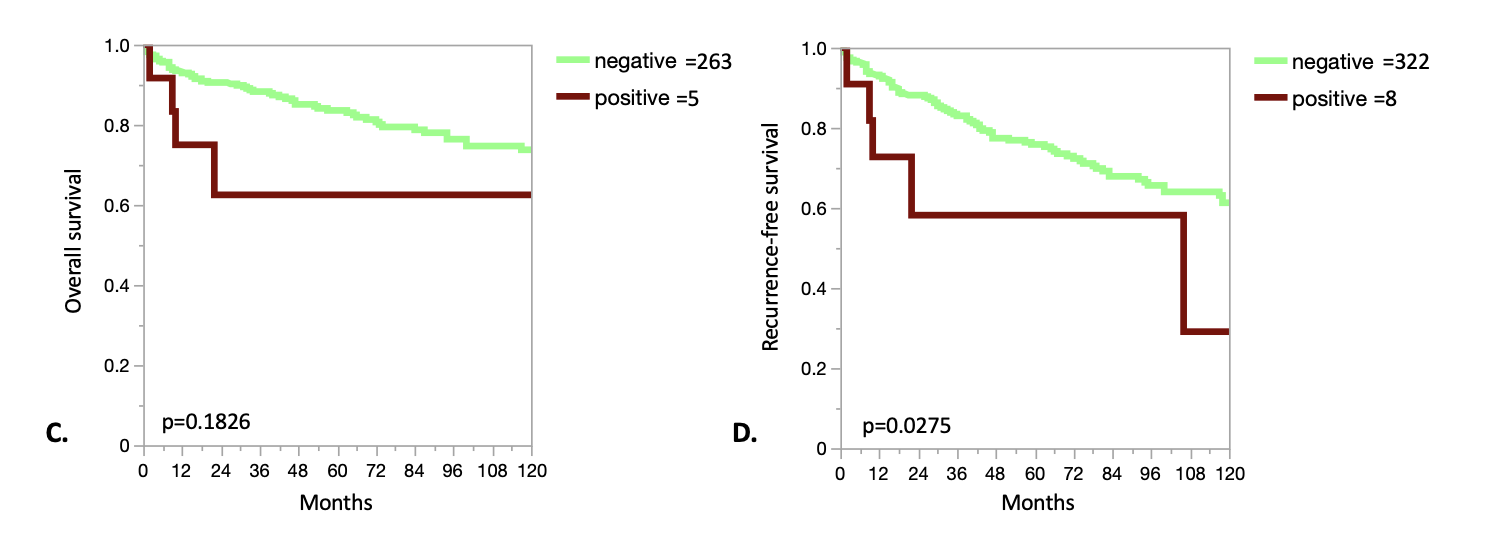
**
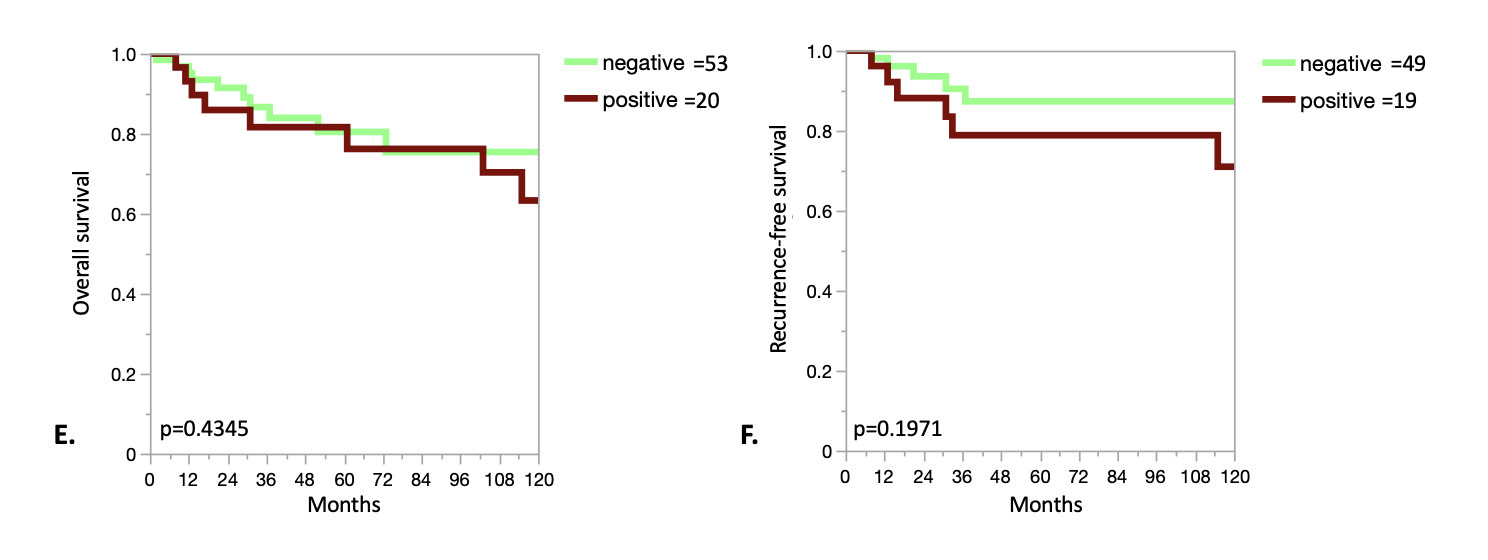
**

**Suppl Fig. 2.**

Prognostic impact of AGR2 immunostaining in different tumor entities. Overall (**A.**) and recurrence-free survival (**B.**) in urothelial carcinomas, overall (**C.**) and recurrence-free (**D.**) survival in clear cell renal cell carcinomas and overall (**E.**) and recurrence-free (**F.**) survival in papillary renal cell carcinomas.
