# Supplementary material for: Prevalence and Significance of AGR2 Expression in Human Cancer
Source: Cancer Med. 2024 Nov 12;13(21):e70407. doi: 10.1002/cam4.70407 (PMC11557986; doi:10.1002/cam4.70407)
Supplement: Supplementary file 2 — Data S2. [file CAM4-13-e70407-s001.docx]

|  | **AGR2 staining pattern of normal tissue** | **AGR2 staining in cancer** | | | | **AGR2 staining in normal tissue** | **AGR2 staining in cancer** |
| --- | --- | --- | --- | --- | --- | --- | --- |
|  |  | **negative (%)** | **weak (%)** | **intermediate (%)** | **strong (%)** |  |  |
| **Esophagus** | mostly absent in squamous epithelial cells | Squamous cell carcinoma of the esophagus | | | | 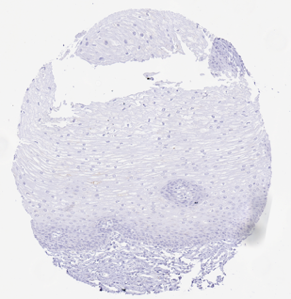 | 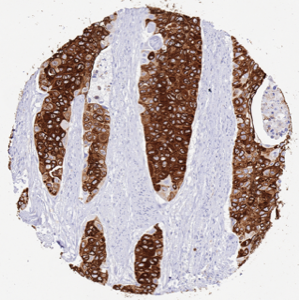 |
|  |  | 35.7 | 17.1 | 25.7 | 21.4 |  |  |
| **Stomach** | strong, predominantly cytoplasmic (except parietal cells) | Gastric adenocarcinoma, diffuse type | | | | 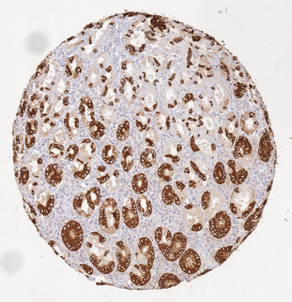 | 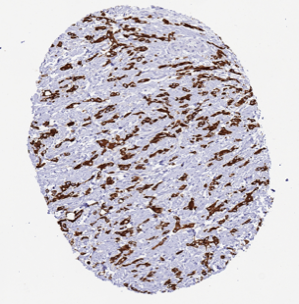 |
|  |  | 1.6 | 0.0 | 0.5 | 97.9 |  |  |
| **Colon** | strong, predominantly cytoplasmic | Adenocarcinoma of the colon | | | | 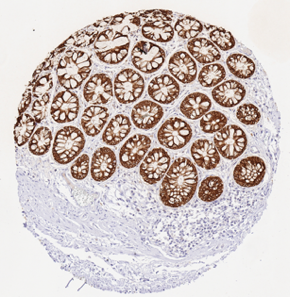 | 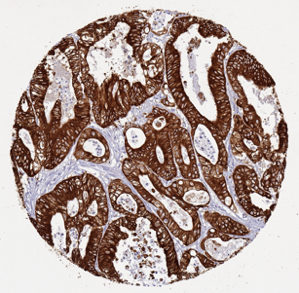 |
|  |  | 0.4 | 0,7 | 1.4 | 97.5 |  |  |
| **Urothelium** | strong, predominantly cytoplasmic | Urothelial carcinoma, pT2-4 G3 | | | | 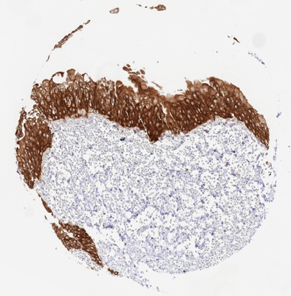 | 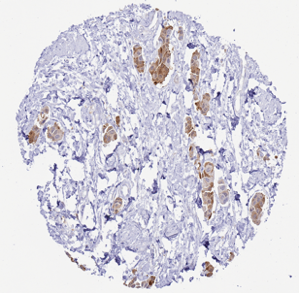 |
|  |  | 19.1 | 11.7 | 13.3 | 55.9 |  |  |

**Suppl. Table 2 Staining pattern and intensity of AGR2 in normal tissues in comparison to tumor tissue**
